# Downregulation of Mcl-1 synergizes the apoptotic response to combined treatment with cisplatin and a novel fiber chimeric oncolytic adenovirus

**DOI:** 10.3892/or.2012.1636

**Published:** 2012-01-16

**Authors:** LIANGSHUN YOU, YING WANG, YINGMING JIN, WENBIN QIAN

**Affiliations:** Institute of Hematology, The First Affiliated Hospital, College of Medicine, Zhejiang University, Hangzhou 310003, P.R. China

**Keywords:** cisplatin, oncolytic adenovirus, synergy, apoptosis, Mcl-1, HeLa cell, HT-29 cells

## Abstract

The aim of this study was to examine the effects of SG511, a novel fiber chimeric oncolytic adenovirus with E1B 55-kDa deleted, combined with cisplatin on cancer cells and to identify their underlying mechanisms. The combined effect of SG511 and cisplatin on HeLa and HT-29 cells was assessed by a crystal violet assay and an MTT assay, followed by combination index analysis. Cell apoptosis was evaluated by DAPI staining and visualized by fluorescein-mediated signal detection. Mitochondrial membrane potential was detected by flow cytometric analysis of Rhodamine 123 accumulation. The activation of the caspase pathway and the expression of Bcl-2 family proteins were examined by western blotting. Results show that SG511 vector infected various human cancer cell lines and induced growth inhibition effectively. Of note, SG511 synergistically enhanced the anti-proliferative activity of cisplatin, a DNA-damaging agent, against HeLa and HT-29 cells *in vitro*, concomitantly with increased apoptosis and activation of the mitochondrial pathway. Furthermore, treatment with SG511 alone or in combination with cisplatin resulted in reduced expression the anti-apoptotic Bcl-2 family member Mcl-1 in HeLa and HT-29 cells. Importantly, this combination did not increase the growth inhibitory effects of cisplatin on human normal liver cells. Collectively, SG511, a novel fiber chimeric oncolytic adenovirus, sensitizes cancer cells to apoptosis by reducing anti-apoptotic Mcl-1 protein levels.

## Introduction

Virotherapy is a tumor-specific strategy, in which viruses engineered to selectively kill tumor cells, through targeted alterations in the cancer such as p53 mutation, viral deletion, tissue-specific transcriptional control or tumor-specific receptors, thus, representing a promising approach for the treatment of neoplastic diseases refractory to conventional therapies (1,2). Conditionally replicating adenoviruses (CRAds) based on the native adenoviral serotype 5 (Ad5) are most commonly used for generation of oncolytic agents that include ONYX-015, a vector with a deletion in the E1B region, and the AdΔ24 vector, containing a 24-bp deletion in the E1A gene (3–5). Recent generation of oncolytic adenoviruses have combined E1A and E1B alterations in an effort to improve the selective killing of cancer cells (6). Although their selective and antitumor activity was demonstrated in numerous experiments with different types of tumors *in vitro* and *in vivo*, the CRAds based on Ad5 has a major limitation to be solved owing to weak expression of coxsackie adenovirus receptor (CAR) in tumor cells. This CAR deficiency makes cancer cells resistant to Ad5 infection (7). To overcome this problem, non-replicating adenoviral vectors containing chimeric type 5 and type 35 or type 11 have been successfully constructed, which permit CAR-independent infection of tumor cell and are used for effective gene transfer (8,9). For oncolytic adenoviral vector, we and others have recently introduced Ad5/35 and Ad5/3 fiber chimeric replicating viruses, and demonstrated their broad antitumor activity regardless of CAR expression in cancer cells (10–13). These data obtained from the strategy of adenoviral tropism modification using alternate cellular attachment receptors predicts an improved therapeutic index for the virotherapy of cancer.

The effect of oncolytic adenoviruses against cancer cells could be significantly improved by combination with standard chemotherapies (2). There are several preclinical studies suggesting enhanced and even synergistic cell killing and anticancer activity when oncolytic adenoviruses and chemotherapeutic agents including cisplatin, gemcitabine, epirubicin, cyclophosphamide, 5-fluorouracil, docetaxel, and temozolomide have been combined (5,14–23). Phase II clinic trails have been undertaken to evaluate the use of ONXY-015 combined with cisplatin and 5-fluorouracil therapy in patients with recurrent squamous cell carcinoma of the head and neck. The results show an improved anticancer effect when compared with historical data in patients treated with either ONXY-015 or chemotherapy alone (24–26). In China, the addition of a modified version of ONXY-015 (H101) to chemotherapy has already entered Phase III trail with promising results (27). Together, these studies on combination oncolytic adenovirotherapy and chemotherapy have shown enhanced and even synergistic antitumor efficacy, suggesting the combination therapy represents a promising strategy for the treatment of cancer; however, the mechanism for the synergy between CRAd virotherapy and chemotherapy is unclear.

In this study, we investigated the effects of SG511 (a new Ad5/11 fiber chimeric oncolytic adenovirus) on human cancer cell lines. Next, we evaluated whether the combination treatment of SG511 plus cisplatin perform robust synergistic killing in tumor cells. We further studied the mechanism of enhanced cytotoxicity induced by the combination therapy with attention to the alteration of Bcl-2 family proteins, because pro- and anti-apoptotic Bcl-2 family proteins dictate the ultimate sensitivity or resistance of cancer cells to various apoptotic stimuli (28).

## Materials and methods

### Cell lines and culture

HeLa (human cervical cancer cell line), HT-29 (human colorectal cancer cell line), SW480 (human colon cancer cell line), Panc-1 (human pancreatic carcinoma cell line), and L-02 (normal human liver cell line) were purchased from the Shanghai Cell Collection (Shanghai, China). Cells were maintained in RPMI-1640 medium (Hyclone Laboratories, Logan, UT, USA) supplemented with 10% fetal bovine serum (FBS; Life Technologies, Inc., Grand Island, NY, USA) and 1% L-glutamine (Life Technologies). The cells were cultured at 37°C in 5% CO_2_ humid atmosphere.

### Human mesenchymal stem cell isolation and culture

Bone marrow mononuclear cells were obtained from bone marrow samples from healthy donors after informed consent, and isolated by Ficoll density gradient and cultured in DMEM medium (Gibco-BRL, Grand Island, NY, USA) containing 10% FBS. After 3 days, non-adherent cells were discarded and adherent cells were replenished with fresh medium twice a week. When the culture reached confluency, mesenchymal stem cells (MSCs) were detached and identified by flow cytometry using the following mAbs: CD29-FITC, CD90-FITC, CD44-PE (all from eBioscience Inc., San Diego, CA, USA), and CD166-PE (from R&D Systems, Minneapolis, MN, USA). MSCs were used for experiments between the 3rd and 4th passages.

### Crystal violet assay

Cells were plated in 96-well or 24-well dishes at 1×10^5^ cells/ml 12 h, and then treated with SG511, cisplatin alone or SG511 combined with cisplatin at a ratio of 10:1. After incubation for 48 h, medium was removed, and dishes were washed with phosphate-buffered saline (PBS) twice, and then stained with 2% crystal violet for 5 min, followed by three rinses with water. Air-dried dishes were photographed. For the detection of OD value, 100 μl 33% acetic acid was applied per well to decolorize. Absorbance was read at 595 nm with a Bio-Rad microplate reader. The experiments were repeated at least three times, each time in triplicate.

### MTT assay

Cells (3×10^3^) were seeded in 100 μl of the growth medium in the presence or absence of increasing concentrations of cisplatin or SG511 in 96-well plates, and cultured at 37°C in 5% CO_2_ for 48 h. A replication-deficient virus (Ad5/11) was used as a control. Ad5/11 and SG511 virus were kindly provided by Professor Qian (Second Military Medical University, Shanghai, China). The 3-(4,5-dimethylthiazol-2-yl)-2,5-diphenyltetrazolium bromide (MTT) assay was performed as previously described (29).

### Evaluation of apoptosis

The treated or non-treated cells at 1×10^5^/ml were washed with PBS, fixed in 4% paraformaldehyde for 30 min, washed with PBS, and then stained with 4,6-diamidino-2-phenylindole (DAPI, 0.5 μg/ml) for 3 min in the dark. After washed with PBS, cells were observed under a fluorescent microscopy (Olympus, Japan) with a peak excitation wavelength of 340 nm.

### Mitochondrial membrane potential measurement

HeLa cells treated with SG511, cisplatin, or SG511/cisplatin combination were collected, and then washed, stained with 5 μg/ml of Rhodamine 123 (Molecular Probes, Eugene, USA) at 37°C for 15 min. Rhodamine 123 was excited with a 488 nm argon ion laser, fluorescence emission was measured at 530 nm using flow cytometry (Becton-Dickison).

### Western blot analysis

Protein samples were diluted with sodium dodecyl sulfate-polyacrylamide gel electrophoresis (SDS-PAGE) loading buffer, boiled for 3 min before loading on a 12% SDS-PAGE gel, and then transferred to polyvinylidene difluoride (PVDF) membranes (Millipore, Bedford, USA). The membranes were blocked with 5% non-fat dry milk and then incubated with corresponding primary antibodies. After incubation with peroxidase-conjugated secondary antibodies (MultiSciences Biotech, Hangzhou, China), immunodetection was done using an enhanced chemiluminescence detection kit (KPL, Baltimore, USA). All primary antibodies were purchased from Cell Signaling (Danvers, USA).

### Statistics

Two-way analysis of variance (ANOVA) was used to determined statistical significance. Results of combined treatment were assessed by calculating combination index (CI) values using CalcuSyn software (Biosoft, Cambridge, UK). According to this method, synergy was expressed as log10 (CI) vs. fraction affected, and log10 (CI) <0 indicates synergy; log10 (CI) = 0 indicates an additive effect; and log10 (CI) >0 indicates antagonism.

## Results

### Characterization and infectivity of SG511 oncolytic virus

**Figure 1:**
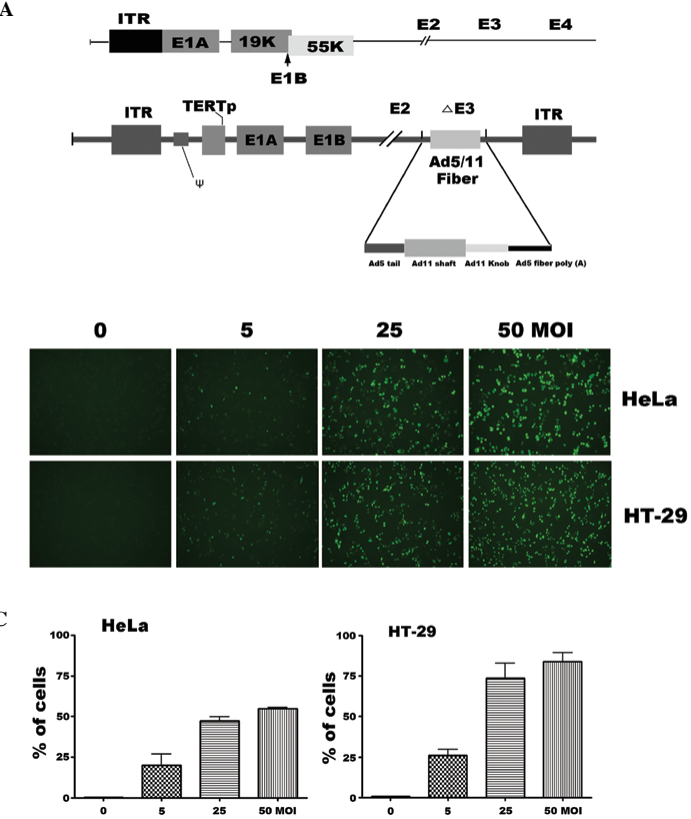
Schematic representation of SG511 virus and infectivity of the viruses in HeLa and HT-29 cancer cell lines. (A) Compared to wild-type Ad5, SG511 has an E1B 55-kDa deletion and an Ad5/11 chimeric fiber in E3 region. (B) HeLa and HT-29 cells were infected with SG511-GFP at the indicated multiplicity of infection (MOI) for 12 h, and observed under a fluorescence microscope to detect GFP (original magnification, ×200). One representative experiment of three performed is shown. (C) HeLa and HT-29 cells were treated with SG511-GFP at MOIs of 5.0, 25, 50 for 12 h. Treated cells were then analyzed using a FACScan flow cytometer and CellQuest software. Three replicates were performed.

The oncolytic adenovirus, SG511, was engineered to delete the E1B-55-kDa, which is similar to ONYX-015, and to have Ad5/11 chimeric fiber (Fig. 1A). Two established human cancer cell lines (HeLa, HT-29) were used to evaluate the infectivity of SG511 vector. After infection with an enhanced green fluorescent protein (EGFP) expressing SG511 virus (SG511-GFP), green fluorescence was analyzed by fluorescence microscopy. Results showed a dose-dependent shift in fluorescence when the cells were treated with SG511-GFP at MOIs of 5, 25 and 50 (Fig. 1B). Next, fluorescence-activated cell sorting analysis was used to quantitate the percentage of GFP-positive cancer cells which indicate the infective capacity of the virus. As seen in Fig. 1C, 53.9% of HeLa cells and 78.4% of HT-29 expressed SG511-mediated GFP 12 h after infection at a MOI of 50.

### Selective broad antitumor activity of SG511

**Figure 2:**
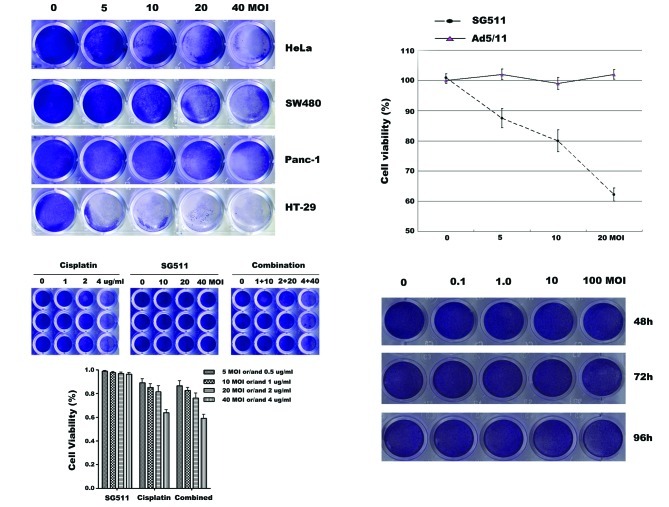
Anticancer activity of SG511 virus and the cytotoxicity to human normal cells when SG511, cisplatin was used alone or in combination. (A) HeLa, SW480, Panc-1, or HT-29 cells were seeded into 24-well plates and treated with the indicated concentrations of SG511. After 48 h, cell viability was assessed by a crystal violet assay. (B) HT-29 cells were treated with SG511 and Ad5/11 vectors, respectively at the indicated MOIs for 48 h. The cell viability was determined by an MTT assay. (C) Human normal liver cells (L-02) were treated with cisplatin, SG511 alone, or two agents together at the dosage indicated for 48 h. Cell viability was assessed by crystal violet staining. The means and standard errors of results from three independent experiments are shown (lower panel). (D) MSCs were infected with SG511 at the indicated MOIs for 48, 72 and 96 h, respectively. Cytopathic effects were evaluated by crystal violet assay.

According to the mechanism of oncolytic virus, the primary character of oncolytic adenovirus is its ability of infecting cancer cells, selectively replicating within them and inducing cell death. To detect the antitumor activity of SG511, HeLa, SW480, Panc-1 and HT-29 cancer cell lines were infected with SG511 at the indicated MOIs. At 48 h after viral infection, cells were stained with crystal violet solution. Results showed that SG511 killed all tested cancer cell lines effectively in a dose-dependent way. Tumor cells were almost complete eliminated when the virus was used at a MOI of 40 (Fig. 2A). Next, we compared the inhibitory effect of SG511 to that of Ad5/11 (a fiber chimeric non-replicating virus) by an MTT assay (Fig. 2B). SG511 induced concentration-dependent cell death in HT-29 cells, whereas Ad5/11 did not cause any detectable cytototoxic effect. We examined effects of SG511 and cisplatin on human normal hepatic cell line L-02. As shown in Fig. 2C, the treatment of cisplatin alone at 2 μg/ml and 4 μg/ml elicited a marked growth inhibition. In contrast, SG511 did not result in obvious cytotoxicity toward normal cells. Furthermore, combined use of cisplatin and oncolytic virus SG511 had only slightly greater cytotoxic effect compared with cisplatin alone. We further investigated the cytotoxicity of SG511 against human normal MSCs. As shown in Fig. 2D, SG511 at the indicated dosage did not apparently affect the viability of human MSCs. These data suggest that SG511 is an ideal tumor-specific replicative adenovirus for virotherapy of cancer.

### SG511 synergistically enhances cisplatin-induced cancer cell death

**Figure 3:**
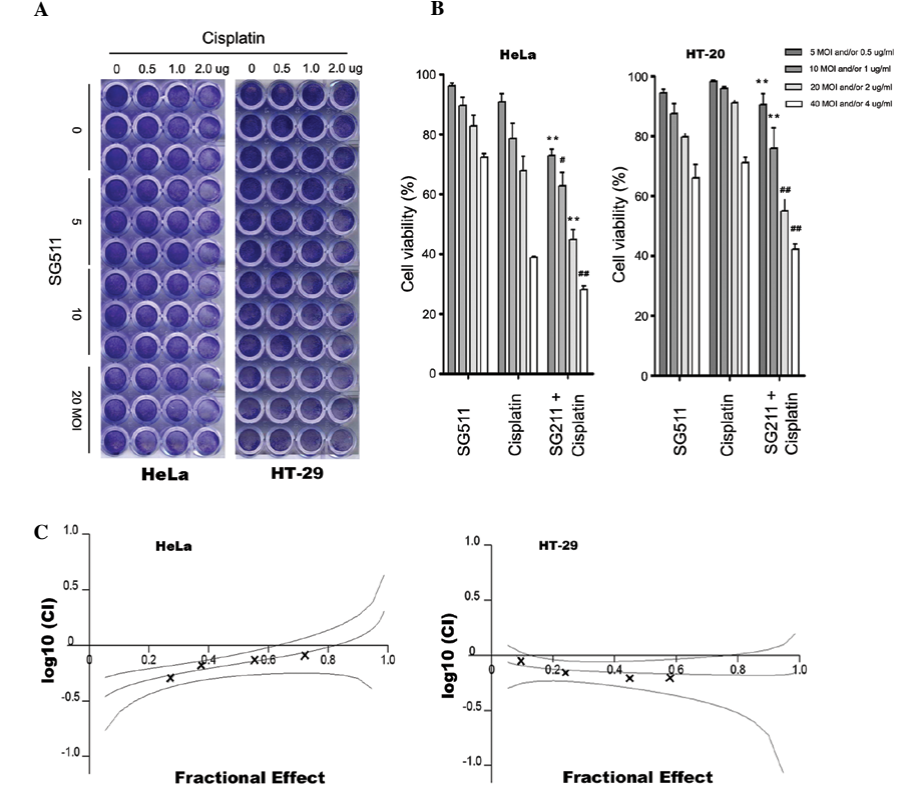
Enhanced suppression of tumor cell proliferation using the combination of SG511 and cisplatin. (A) Human cancer cell lines (HeLa and HT-29) were treated with SG511, cisplatin, and SG511-cisplatin combination. Cell viability was determined by a crystal violet assay. The image shown is representative of three independent experiments. (B) Graph demonstrates the results of MTT assays with drugs used in combination, and shows average values from three independent experiments, bars represent standard deviation. ^#^P<0.05; ^**^P<0.01; ^##^P<0.001 (combination vs. cisplatin). (C) Synergy was quantified by combination index (CIN) analysis and expressed as log10 (CIN) vs. fraction affected. Where calculable, 95% confidence intervals are shown.

We treated cancer cells with low concentrations of cisplatin in combination with oncolytic virus SG511. HeLa cells and HT-29 cells was infected with SG511 at MOI ranging from 5 to 40 in 96-well plates and then treated with various concentrations of cisplatin (0.5–4 μg/ml). Cell survival was determined at 48 h by crystal violet assay and MTT assay. As shown in Fig. 3A and B, cell death induced by SG511 in combination with cisplatin was significantly enhanced. In particular, when used alone, cisplatin at 4 μg/ml induced 27.8% cell death, SG511 at 40 MOI induced 21.3% cell death. However, when 4 μg/ml cisplatin was combined with 40 MOI SG511, the cell death increased to 72.1% in HeLa cells. Similar results were observed in HT-29 cells. This enhanced cytotoxicity of the combination therapy was confirmed by MTT assay (data not shown). The CI at effective concentration (ED50) were 0.68 for HeLa and 0.69 for HT-29 cells (Fig. 3C), suggesting that SG511 and cisplatin cotreatment is highly synergistic.

### Effect of the SG511/cisplatin combination in cell apoptosis

**Figure 4:**
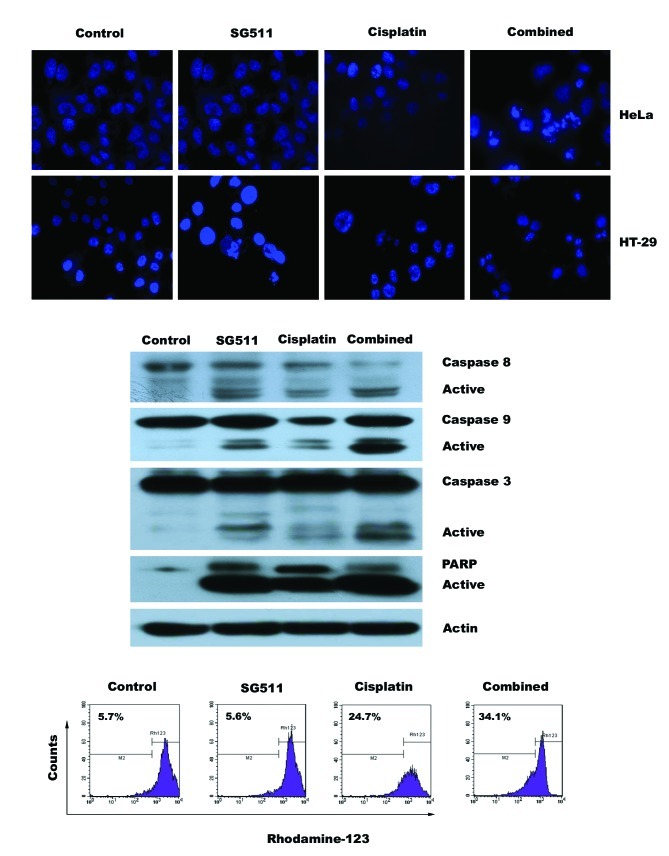
SG511 virus sensitizes cancer cells to apoptosis induced by cisplatin. (A) HeLa and HT-29 cells were treated with SG511 (20 MOI), cisplatin (2 μg/ml), and two-agent combination for 48 h. PBS was used as a negative control. The cells were then fixed and nuclei were stained with DAPI solution. (B) After 48 h, control and the treated cells (HeLa) were harvested, and then cell extraction was subjected to western blot analysis with antibodies against caspase-8, -9, -3 and PARP. Actin was used as a loading control. (C) HeLa cells were incubated with Rhodamine 123 dye, followed by analysis in a FACScan flow cytometer. Representative of three separate experiments.

To study a possible mechanism that contributes to the enhanced cytotoxicity induced by the combination of SG511 and cisplatin, we evaluated whether this combination therapy results in increased apoptosis in cancer cell lines. As shown in Fig. 4A, each agent administered individually exhibited only slight apoptosis evidenced by fluorescence microscopy analysis after DAPI staining, whereas a higher number of condensed and/or fragmented nuclei in HeLa and HT-29 cells was observed when SG511 was used in conjunction with cisplatin. We then examined the effect of combined therapy on caspase activation in HeLa cells. Consistent with the above findings, combined but not individual treatment results in a pronounced increase in cleavage of caspase-3 and caspase-9 and degradation of poly (ADP-ribose) polymerase (Fig. 4B). Furthermore, increased loss of mitochondrial membrane potential (ΔΨm) was observed in cells treated with the combination, evaluated by flow cytometry after staining with the Rhodamine 123 dye (Fig. 4C). Altogether, these findings demonstrate that the combined treatment with low concentrations of SG511 and cisplatin results in synergistic cytotoxicity by inducing apoptosis of both the HeLa and HT-29 cancer cells.

### Mechanism of enhanced apoptosis induced by the combination therapy

**Figure 5:**
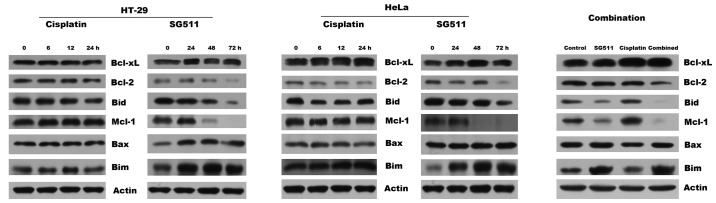
Effect of SG511 alone, or combined with cisplatin on Bcl-2 family member proteins in cancer cells. HeLa and HT-29 cells were either not treated or treated with SG511 (40 MOI) and cisplatin (4 μg/ml), and the combination for the indicated time points. Cells were harvested and whole cell extracts were subjected to western blot analysis with antibodies against Bcl-xL, Bcl-2, Bid, Mcl-1, Bax, Bim and actin. The data are representative of three determinations with identical results.

We investigated the potential molecular mechanism of sensitization of cancer cells to SG511/cisplatin combination, by examining possible alterations in the expression levels of proapoptotic and antiapoptotic signaling molecules. As observed in the western blots (Fig. 5), individual treatment with cisplatin at 4 μg/ml did not induce discernible changes in the expression of Bcl-2, Bid, Mcl-1, Bax, and Bim in HT-29 and HeLa cells. In marked contrast, treatment with SG511 at a MOI of 40 downregulated the levels of multidomain anti-apoptotic proteins Bcl-2, and Mcl-1, and upregualted the level of pro-apoptotic Bax. Cleavage of BH3-only pro-apoptotic protein Bid and Bim accumulation was also observed in cells treated with SG511. These conformational changes were also observed in HeLa cells after treatment with SG511 combined with cisplatin. However, these two agents, alone or in combination, had no effect on the expression of Bcl-xL in the cancer cell lines.

## Discussion

The antitumor potency of an oncolytic adenovirus is largely dependent on the capacity of the virus to infect target cells (7,30,31). Recently, it has been demonstrated that chimeric Ad5 vector possessing fiber proteins derived from group B adenoviruses such as Ad35, Ad11 can efficiently enter tumor cells that are refractory to Ad 5 infection by utilizing CD46 as a high-affinity primary attachment receptor (32). Previous studies suggest that CD46, a membrane regulator of complement activation, is overexpressed on human malignancies including colorectal, cervical, and pancreatic carcinomas compared with their normal counterparts (33–36). In this study, we used human colorectal, cervical, and pancreatic carcinoma cell lines, and demonstrated that SG511, a new Ad5/11 fiber chimeric oncolytic adenovirus, efficiently infected various tumor cell lines and led to cytopathic killing of cancer cell *in vitro*, without damage to human normal liver cells. Together, these results suggest that SG511 is a new targeted oncolytic adenoviral vector with broad antitumor activity.

Cisplatin, a well known DNA-damaging agent, has broad spectrum of activity against epithelial cancers and has become the foundation of curative regimens in testicular and ovarian cancers. It also demonstrates significant activity against cancers of the lung, head and neck, esophagus, bladder, cervix and endometrium (37,38), however, drug-resistance and severe toxicity present major hurdles associated with cisplatin therapy. An excellent example to highlight this limitation is with ovarian cancer, which generally responds well to cisplatin-based chemotherapy. Unfortunately, the initial response is not durable, and tumors in most patients become resistant to the drug (29,39). Earlier clinical trails have indicated that oncolytic adenoviruse therapy combined with cisplatin elicit greater antitumor efficacy in the patients with head and neck or esophagus cancer, without an increase in toxicity. Recently, some studies have shown that the use of cisplatin conjugated with oncolytic adenoviral vectors results in an apparent synergistic cytotoxicity in human hepatocellular, nasopharyngeal, lung adenocarcinoma, and ovarian, colorectal, cervical cancer cells (23,40–42). Consistent with those results, our *in vitro* data showed that SG511 alone has marked antitumor effects on HeLa, SW480, Panc-1, and HT-29 cells at a MOI of 40, whereas modest to mild effects were observed at a MOI of 20 or lower. Interestingly, SG511 did have a sensitizing effect on HeLa cells to cisplatin. Similar sensitizing effect was also observed in HT-29 cells that are relatively resistant to cisplatin. Since classical apoptosis has been presumed the mechanism of adenovirus-induced cell death (43), we investigated the role of apoptosis in cytotoxicity resulting from the combination therapy. The data showed that the nuclear morphology of cells treated with SG511 combined with cisplatin exhibited increased apoptosis characterized by condensed and/or fragmented nuclei. SG511 in combination with cisplatin elicited a more pronounced activation of caspase-9, -3, and PARP. Furthermore, this combination therapy resulted in a high level of ΔΨm loss. These data suggest that SG511 sensitizes cells to cisplatin through activation of caspase-9 and induction of apoptosis.

Apoptosis is regulated in part by the Bcl-2 family of proteins which consist of both proapoptotic (Bax and Bak) and antiapoptotic (Bcl-2, Bcl-xL, A1, Mcl-1 and Bcl-w) proteins (44). In cancer, antiapoptotic members are often overexpressed, rendering cancer cells resistant to apoptosis (45). Our data show that oncolytic virus SG-511 strongly sensitizes human cervical and colorectal cancer cells to apoptosis induced by cisplatin. Furthermore, treatment with SG511 alone or combined with cisplatin induces increases in the levels of Bim and Bax, and actives Bid. Direct activator BH3-only proteins, such as Bid and Bim, have been reported to activate Bax and Bak at the outer mitochondrial membrane leading to cytochrome c release which seems to be primarily responsible for ΔΨm loss (46,47). In this study, the combination therapy results in increased ΔΨm loss and caspase-9 activation, supporting this view. Mcl-1 is a multidomain antiapoptotic member of the Bcl-2 family that acts through several mechanisms to block mitochondrial outer membrane permeabilization and apoptosis (48). For example, Mcl-1 can interact with truncated Bid and inhibits its ability to activate the mitochondrial death pathway (49). Our data show that cisplatin do not affect level of Mcl-1 in HeLa and HT-29 cells. Whereas, SG511 produced a marked decrease in the levels of Mcl-1, a short-lived protein critical for cancer cell survival (50,51). It was reported that downregulation of Mcl-1 induced by cycloheximide results in acceleration of apoptosis in HeLa cells (52). Moreover, previous studies indicate that overexpression of Mcl-1 in human cervical and colorectal cancer tissues is associated with a poorer prognosis (53). These data suggest that Mcl-1 downregulation by SG511 virus contributed significantly to potentiation of cisplatin lethality in these cancer cells and to the resulting synergistic antitumor interactions.

In summary, our results demonstrate that SG511, a new fiber chimeric oncolytic adenovirus, can infect and kill various cancer cell lines effectively. We show that SG511 combined with cisplatin increases apoptosis and cell death in tumor cells without enhanced lethality in normal cells. Additionally, SG511 effectively downregulate expression of Mcl-1 and Bcl-2 genes, which may contribute to activation of the mitochondrial pathway for apoptosis. Thus, we believe that the use of SG511 should be an attractive strategy to improve the therapeutic results of cisplatin-based chemotherapy of cancers.
